# The Nation's Health. The Stamping out of Venereal Disease

**Published:** 1917-12

**Authors:** 


**The Nation's Health. The Stamping out of Venereal Disease.** By Sir MALCOLM MORRIS, K.C.V.O. Pp. viii. 152. London: Cassell & Company Ltd. 1917. Price 3s. 6d. net.

A work on venereal diseases, with special reference to their bearing on public health, and intended particularly for members of County and Borough Councils, Boards of Guardians, lay members of Boards of Management of Hospitals, and Headmasters and Headmistresses, calls for special qualifications in authorship. It requires a thorough mastery of the subject in its scientific aspects, a statesmanship in presenting the national health relationships, and ability to bring forward and present clearly the relative merits of the two sides on controversial points. We think Sir Malcolm Morris has succeeded admirably in the task he set out to accomplish. In this small book the majority of medical practitioners will find much to learn, while those for whom the book is written will find practically all they need know for a thoroughly sound grasp of the subject dealt with.

